# The impact of co-national networks on asylum seekers’ employment: Quasi-experimental evidence from Germany

**DOI:** 10.1371/journal.pone.0236996

**Published:** 2020-08-04

**Authors:** Felix Stips, Krisztina Kis-Katos

**Affiliations:** 1 University of Göttingen, Göttingen, Germany; 2 University of Stellenbosch, Stellenbosch, South Africa; 3 University of Göttingen and IZA, Bonn, Germany; University of Luxembourg and Luxembourg Institute of Socio-Economic Research (LISER), LUXEMBOURG

## Abstract

Using novel registry data on persons receiving asylum welfare benefits in Germany for the period from 2010 to 2016, and quasi-experimental variation induced by German allocation policies, we identify the role that the size and composition of local co-national networks of asylum seekers play for formal labor market access within the same group. While the individual employment probability is not linked to network size, it increases with the number of employed local co-national asylum seekers and decreases with the number of non-employed network members, thereby underlining the central importance of network quality. JEL Classification: F22, J61, R23.

## Introduction

The large influx of asylum seekers to Germany at the height of the so-called ‘European refugee crisis’ brought the issue of economic integration of refugees back to the heart of policy debates in Germany. In only two years (2015 and 2016), more than 1.2 million new asylum seekers registered in Germany, which was more than what Germany has experienced over the previous fifteen years (from 2000 to 2014, [1]). This dynamic has also resurrected the interest in the economic consequences of geographic agglomeration of asylum seekers and refugees and triggered policy changes, for instance in the form of the 2016 *Integration Act*. This new legislation introduced restrictions of residence not only for asylum seekers but also for recognized refugees for a period of up to three years, with the explicit intention to prevent agglomeration of refugees in certain areas [2]. However, given that such restrictions of movement pose severe limitations on the individual rights of migrants, context-specific evidence on the effects of a regional agglomeration of co-national asylum seekers is crucial in order to inform future policy making.

Empirical studies have addressed this issue before, among others, by exploiting refugee dispersal policies to identify the causal effects of the resulting so-called `ethnic enclaves’ [3]. They often yield positive results: ethnic networks tend to increase job participation and earnings [4], and more strongly so for less-skilled migrants [5]. The main driver of these positive results is network quality: ‘high-quality’ networks have strong positive effects, while ‘low-quality networks’ no or even negative impacts [6]. This is in line with endogenous network models, where employed members are able to share job-relevant information with non-employed members within the network, thereby increasing their employment chances [7–10]. Taking dynamic considerations into account, information effects within the network may also depend on the size of networks as well as experience of network members in the destination country [9–10]. Empirical evidence on these latter channels still remains limited.

This paper re-investigates these hypotheses using a hitherto unused large administrative data source on asylum welfare benefit recipients in Germany, available throughout the years 2010 to 2016, and focusing on co-national networks of asylum seekers. Asylum seekers–who are not yet recognized as refugees–are an interesting subpopulation to study as they usually face the most severe limitations to enter the labor market but at the same time their early labor market experiences are often argued to play a crucial role in their social and economic integration [11, 12]. During our analyzed time period, on average about 3% of male asylum seekers in Germany were employed, although with substantial heterogeneities across time, space and by nationality [13]. In this paper, we focus on the question of whether the presence of further co-national asylum seekers within the same local labor market can help or inhibit early labor market entry of male asylum seekers. As such early labor market access is possible among many circumstances but requires cumbersome procedures, other co-national asylum seekers may provide informational advantages, but at the same time may also act as direct competitors or crowd-out information.

Our identification strategy relies on the substantial idiosyncratic variation in the assignment of asylum seekers to German counties that is accompanied by a so-called ‘domicile obligation’, which requires asylum welfare benefit recipients to stay in their assigned county. The same dispersal policy has also been exploited for investigating the effects of the spatial allocation of asylum seekers on regional outcomes in Germany [14–16]. In order to capture variation in local labor market conditions as well as national policy dynamics, we additionally rely on an extensive set of fixed effects, focusing on within-variation. We relate the probability that an asylum seeker participates in formal employment to the size of the local co-national network of asylum seekers, which we measure by the number of other co-national asylum seekers’ welfare benefit recipients residing in the same county. Our empirical model specification controls for a full set of county-year, nationality-year, and nationality-county pair fixed effects. By that we identify network effects by comparing asylum seekers of different nationalities residing within the same county and in the same year, after having factored out all variation in general local labor market characteristics, nationality-specific fluctuations over time as well as the time-invariant effects of county-specific historical migrant networks.

Our results indicate a small and insignificant link between a larger presence of co-national asylum seekers and the individual probability of employment in the fully specified model. Decomposing the network measure along various dimensions, we find results in favor of the network quality model as the probability of individual employment increases with the presence of more employed co-nationals and decreases with additional unemployed co-nationals living within the same county. When focusing on cohorts of previously arrived asylum seekers, we do not find strong evidence for cohort effects, although these results are subject to the limitation that we are only able to measure variations in the number of local asylum seekers but not the number of officially recognized refugees. Additionally, we provide some further evidence on the relevance of the age composition within the network. Even after controlling for the age of the individual asylum seeker, having more co-national youth within the same county reduces employment chances, whereas a relatively larger presence of older co-nationals is positively linked to individual employment. The results on part- and full-time employment are less conclusive but suggest to some degree that larger (and better quality) networks may primarily ease transition into part-time employment.

Our study is closely linked to the literature that uses refugee dispersal policies to investigate the impact of differing migrant densities on the labour market outcomes of refugees [4, 5, 10, 17]. Our identification strategy is most closely related to the strategy used by [10], who focuses on labour market outcomes of refugees in the time after their arrival, without taking the possibility of later spatial re-sorting into account. The implicit assumption behind this strategy is that a relatively shorter time horizon or relatively more rigid movement restrictions limit the ex-post sorting of asylum seekers. In contrast, studies covering longer time horizons usually address ex-post sorting empirically by instrumenting the current network size with historical numbers of allocated asylum seekers [4, 5, 17].

We contribute to the literature by (i) adding new results on the most recent wave of asylum seekers in Germany, evidence on whom is still limited, (ii) specifically addressing asylum seekers, a migrant population that has not been studied quantitatively in this context so far, although being potentially important for understanding early labor market access and integration, and (iii) comparing results based on classical network size and quality models to a dynamic cohort model as proposed by Beaman [10].

A limitation of our study arises from us only being able to measure the number of asylum seekers within any locality over time, and not increases in the number of accepted refugees (although our strategy controls for variations in the number of historical migrants). We thus may underestimate the benefits arising from the presence of previously established cohorts of co-national asylum seekers. However, our study is still able to speak to the policy question of the short-term effects of co-location of asylum seekers for their early labor market access.

### Institutional background and hypotheses

**Fig 1:**
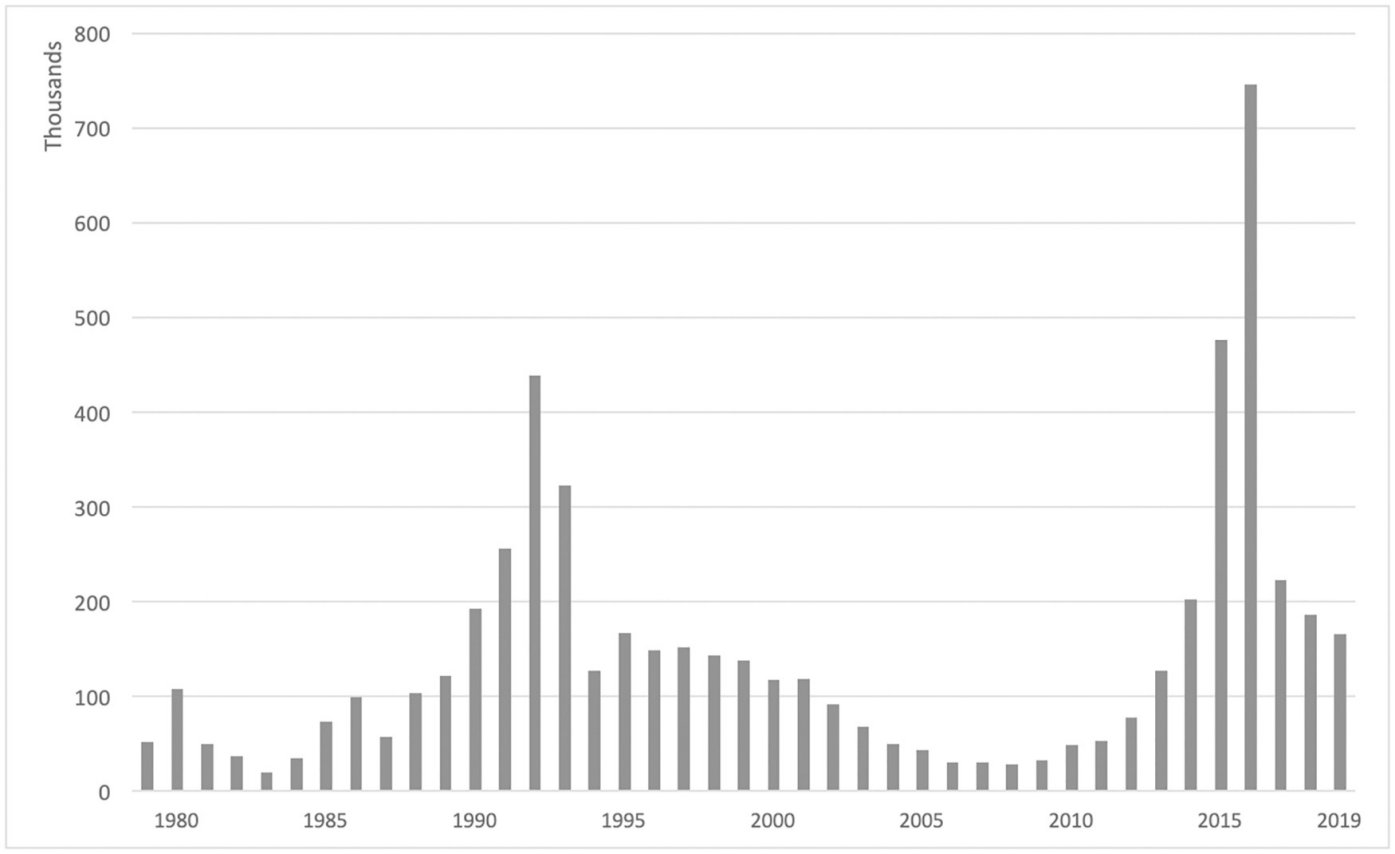
New asylum applications in Germany by year (1979–2019). Source: [1].

Over the last forty years, there were two substantial peaks of inflows of asylum seekers to Germany. A first peak came after the end of the cold war and the accompanying disintegration processes within the Soviet bloc, but this was substantially surpassed by the second more recent wave of the ‘refugee crisis’ that peaked in 2015 and 2016 (see Fig 1). Within just two years, the German asylum system received more than 1.2 million new applications and was facing substantial challenges to process and accommodate the new arrivals. The assignment of asylum seekers followed a set of pre-determined policies and tried to circumvent spatial self-selection. Nonetheless, we do not have enough evidence that would allow us to claim that the location of asylum seekers within the country is fully exogenous. Especially the allocation of asylum seekers from smaller nationalities has also mirrored administrative capacities to deal with certain origin countries (based on e.g., language skills or experience of officials) [12]. Instead, we argue that refugee allocation in Germany has a substantial idiosyncratic component, which together with an extensive set of fixed effects will allow us to study the effects of the size of co-national networks of asylum seekers.

### Institutional background

**Fig 2:**
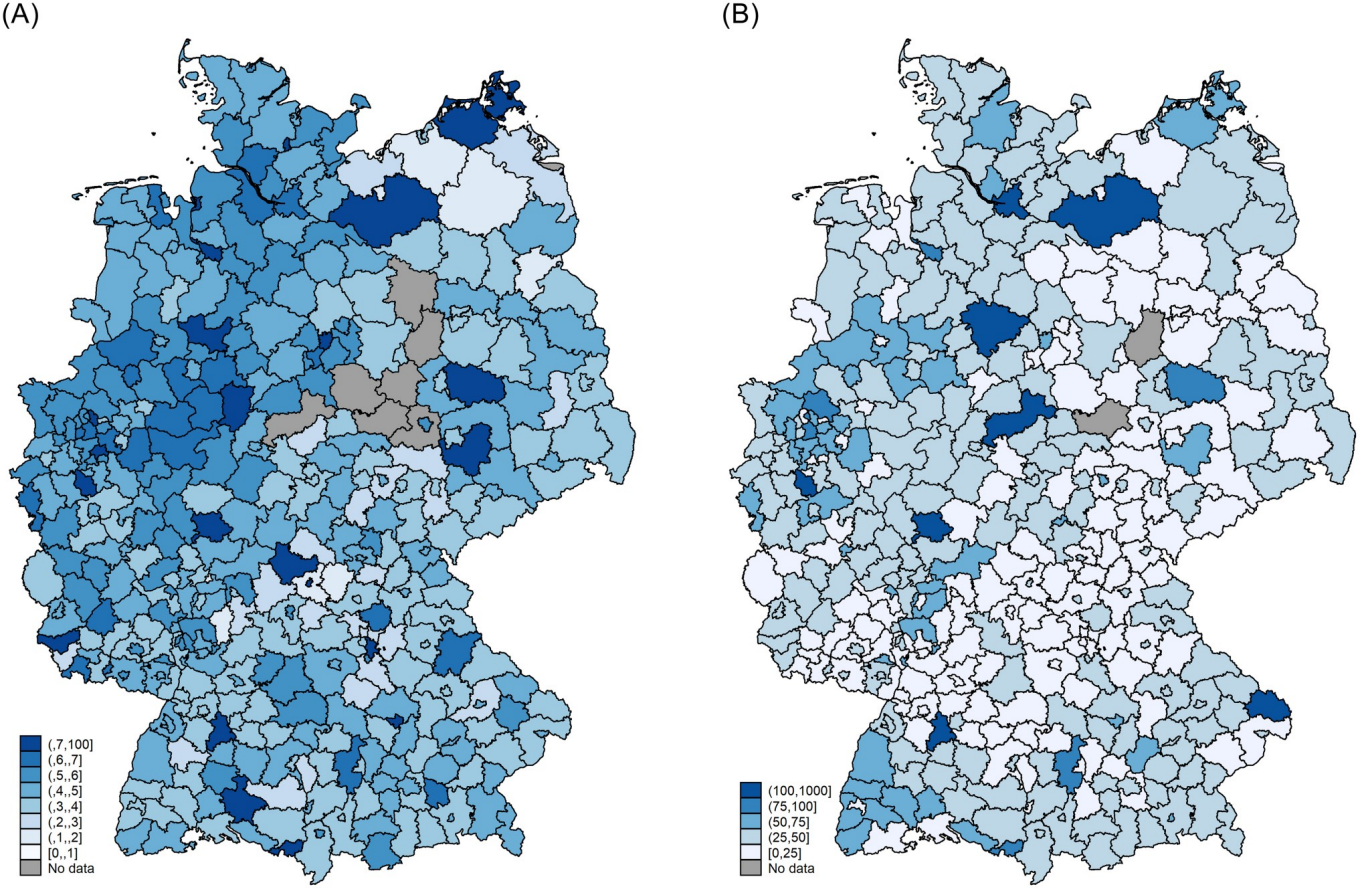
Geographical variation in local asylum seeker networks. Panel A: Share of asylum seekers in total population. Panel B: Asylum seeker network size. Source: [13], own calculations. Notes: Maps show means over the period of observation. Total population is based on Census 2011. Data for counties in grey is not displayed due to confidentiality reasons.

Due to the federal political architecture of Germany, asylum seekers are allocated to different localities in a multi-stage procedure involving different administrative actors. First, all asylum seekers are required to file a formal request in a nearby reception centre of the Federal Office for Migration and Refugees (BAMF). Their data is registered through the electronic EASY system (‘*Erstverteilung von Asylbegehrenden’*) after which applicants are distributed to first reception centres (‘*Erstaufnahmeeinrichtungen’*) in each federal state [18]. In order to spread financial burdens equally across states, the responsible office allocates the number of asylum applicants to each state based on a formula (the so-called *‘Königssteiner Schlüssel’*), which is calculated annually based on state population shares (with a weight of two thirds) and relative tax revenues (with a weight of one third), each from the penultimate year [19]. Additionally, allocation decisions at the state level also take into account current housing availability as well as the nationality of asylum seekers [19, 20]. The latter may be explained by the fact that the ministry’s asylum offices tend to specialise in processing applications from certain countries of origin. This is particularly the case for countries with lower numbers of asylum applicants. For example, in 2017, applications from Sudan were only processed in Lower Saxony, North Rhine-Westphalia, and Rhineland-Palatinate, while Syrian applicants could be processed in all federal states. To accommodate for the specialisation of the offices, dispersion clusters smaller nations to certain states. This process, however, appears to be fairly random and driven in most parts by the expertise of the offices’ staff [12]. As such, in practice the distribution of asylum seekers across German federal states largely mirrors the respective population distribution [12, 14]. Panels A and B of Fig 2 support this view. The average share of asylum seekers receiving asylum welfare benefits compared to the county population size is below 1% for all counties. Overall, the differences between counties in average asylum seeker network size are relatively small and there appears to be no clear pattern in their spatial distribution.

At the state level, asylum seekers initially reside in first ‘reception centres’ for a period of at least six weeks and up to six months (the maximum waiting period was increased from previously three months in 2015) [18]. During this time, they fall under a residence obligation, which restricts their freedom of movement to an assigned district or state, and also face a general work prohibition (except for so-called ‘work opportunities’ inside the ‘reception centres’). This initial waiting time for labour market access was shortened by two legislative changes in 2013 and 2014 from originally twelve to nine and subsequently three months, but lasts at least as long as asylum seekers reside in first reception centres [20].

After the waiting period, asylum seekers are re-assigned according to federal legislation which varies to some degree across states. Most states employ a two-stage procedure whereby applicants are first assigned to counties and subsequently to municipalities, while some assign asylum seekers directly to municipalities. The distribution of asylum seekers to each administrative unit follows various yet structurally similar procedures in each state [21]. By far the most common approach, adopted by nine out of sixteen state authorities, is to fix a distribution key at the county level that is directly proportional to relative population shares. In North Rhine-Westphalia, the population criterion is added with an area measure, while Berlin engages in a consultative process between state and civil society organisations [22]. The remaining states, Thuringia, Bremen, Schleswig-Holstein, and Bavaria employ fixed quotas assigned by decree. These quotas remained largely unchanged over our period of observation. While asylum seekers can submit a formal request to be (re-)allocated to another county, no legal right guarantees that their wishes are taken into consideration, except in the case of family reunion [23]. Also, acceptance rates are generally low and claims are granted only in very exceptional circumstances [20]. Thus, although procedures vary between states, they share a common feature: allocation is very rarely linked to individual wishes, economic prospects, cultural proximity, or even the capacity of municipalities to host asylum seekers [19]. Empirically, [14] confirm this when they find no significant correlation between local socioeconomic characteristics and the number of asylum seekers at the county level.

Allocation policy in Germany did not only have the aim to spread financial obligations evenly but also to prevent the agglomeration of foreigners in certain areas. To achieve this goal, the allocation within each federal state is accompanied by a legislative restriction on the freedom of movement of asylum seekers. The domicile obligation (‘*Wohnsitzauflage*’) confines the place of residence of asylum seekers to their assigned district, while the residence obligation (‘*Residenzpflich*t’) imposes restrictions on movements outside this district [14, 23]. An infringement of these regulations entails an administrative offence including a monetary fine and, in case of repeated occurrence, negative consequences for the prospects of receiving a permanent residence permit [24]. Throughout the period of observation, there have been a number of legislative changes, a detailed depiction of which goes beyond the purpose of our study. A series of liberalizations at the state-level in the period between 2010 and 2014 increased the freedom of movement [16], which in 2015 resulted in an abolition of the residence obligation for applicants with high prospects of staying [2]. Importantly for the identification strategy in this paper, these changes however only applied to those who are not receiving welfare benefits according to AsylbLG [24]. In 2016, these relaxations were quickly revoked with the enactment of the *Integration Act*. Under this act, the domicile obligation may even be applied to approved refugees for a period of one to three additional years [25].

While a general employment ban applies to all new arrivals who still live in ‘reception centres’, this ban is relaxed once asylum seekers have passed the first waiting period and have been resettled to a permanent location. To reflect this strict limitation, we exclude all asylum seekers from our empirical analysis who still reside in first reception centres as they are legally prohibited from entering employment (although we do include them when constructing our asylum seeker network measure).

After re-settlement, employment becomes possible, although still remains subject to a series of conditions and requires the completion of certain procedures. In the first 15 months after their registration, asylum seekers who originate from places that are not considered as ‘safe countries of origin’ are required to request a work permit from the Foreigners’ Office and cannot become self-employed or work for a temporary work agency. In theory, the Foreigners’ Office has the obligation to assert that an appropriate wage is paid and that no EU citizen is willing to accept the same job [26]. In practice, however, the vast majority of employment contracts were approved by the Foreigners’ Office [12].

Further exceptions apply for asylum seekers with an officially recognised job training in a sector with a shortage of skilled workers, for whom no priority checks are required and even the residence restriction can be relaxed [27]. Since late 2016, priority checks have been officially abandoned for all groups, even though not all employment offices were following suit [12]. Anecdotal evidence further suggests that in general, authorities tended to have a positive attitude toward employment of asylum seekers and tended to take benevolent decisions [28]. As an exception from this rule, asylum seekers from so-called ‘safe countries of origin’ who arrived after August 2015 became excluded from entering employment during the entire asylum process. Our empirical strategy will control for such common nationality-specific shifts in labor market access by including a full set of nationality-year fixed effects.

No legal restrictions are made on maximum income or working hours, although welfare benefits and housing costs will be deducted from the income. The allowance rate for these deductions is currently set at 25% of net income or a maximum of 60% of the asylum welfare rate [29]. At latest after the 15th month since arrival, or if the asylum procedure results in granting refugee status or subsidiary protection, all employment restrictions are lifted and applicants can participate normally in the labour market. Rejected applicants may apply for work permits as long as they stay within the country, but have to undergo the Foreigners’ Office priority checks [26].

### Hypotheses

We focus on a specific subset of migrants, asylum seekers, and link their employment outcomes to local co-national asylum seeker networks. Asylum seekers are a special group as they are on average more vulnerable and face larger administrative barriers to their labour market access as general migrants do. Our approach is informed by a rich literature that addresses the broader issue of migrant networks [6, 30], and emphasizes the positive role such networks play in access to employment and income of migrants. The common argument embedding these optimistic findings is that networks are more important for migrants relative to ‘natives’ as their alternatives to finding jobs are more restricted. These restrictions may be viewed in terms of labour market discrimination, ease to access job market information or of disadvantages in host country specific human capital. The marginal utility of using social ties for employment search will in turn be higher [31]. A number of studies provide evidence that confirm lower-skilled and vulnerable migrants select more often into ethnic enclaves [4, 5, 32, 33] and may experience higher returns from living in enclaves [5, 33]. Endogenous sorting thus represents the key identification issue in the literature on the economic returns of ethnic networks, and commonly addressed exploiting quasi-experimental methods.

Conceptually, the literature differentiates between the information channel and the social norms channel of ethnic networks [34]. The information channel stipulates that relevant information is shared with members of the network, but not with outsiders. This may be achieved indirectly by the provision of information about employment opportunities and administrative procedures or directly by the referral of candidates to employers [35]. From a search-model perspective, all factors are equivalent to an exogenous increase in the job arrival rate, which increases the likelihood of accepting a random job offer [36]. Following the canonical setup in [8], the arrival rate can also be considered to endogenously depend on network quality, for example, the employment rate of other members in the network.

The transmission of job-related information through the network also depends on network size [9–10]. In a general equilibrium setting, a larger network may mean more information, but also more competition from similar applicants. For example, [10] develops a dynamic search model of competition for job-relevant information within the network. In each period, agents receive random job offers, which they pass on to other members of the (ethnic) network if they are currently employed, thereby increasing job chances of non-employed members of the network. Exogenous increases in the number of migrants lead to increased competition for this information within the social network and thus lead to an initial negative shock to employment probabilities. Over time, this effect is dampened, and even reversed as an increasing number of migrants enter the labour market, which increases the overall quantity of valuable information.

The social norms channel highlights the importance of peer groups to affect individual decision-making. Labour market access may be affected directly through changes in work ethics, entrepreneurship attitudes, or labour division within the household [37], or indirectly through impacts on the fertility rate and human capital accumulation [5], and in particular the accumulation of host country specific skills [38]. Recently, [39] develop and empirically confirm a two-period search model that focuses on the trade-off between human capital investments and job searches of migrants in Germany, where a larger co-ethnic network implies higher chances of finding a job through the informal (network) channel in the short run, which in turn decreases incentives to invest into human capital and leads to job-skill mismatches and lower wages in the longer run.

In summary, even if we assume that the role of asylum seeker networks does not substantially differ from that of other migrant networks, our expected effect of such networks on the labour market participation of asylum seekers remains theoretically ambiguous. The net effect of networks in the short run is a priori unclear. It arises from increases in the quantity of information (increasing the likelihood of employment), from more competition for information (with a negative effect), and ambiguous social norms effects. In the long run, a potentially negative human capital accumulation effect may also arise. These effects can be expected to vary with the quality and size of the network, as well as with the characteristics of asylum seekers. Network quality is usually measured in form of income, education, or (self-) employment rates of the network members [4, 6] and can be expected to yield more positive net effects in the short run. Similarly, in line with Beaman’s model [10], we would expect to find significant heterogeneities based on the duration of stay in Germany, i.e., a relatively higher presence of more ‘experienced’ co-national asylum seekers in the same county should have (more) positive impacts as compared to a higher presence of recent co-national arrivals.

## Empirical strategy

### Data

We use a novel administrative dataset which combines the Statistic on Asylum Benefits and the Statistic on Special Asylum Benefits for the years of 2010 to 2016 [13]. The pooled yearly cross sections cover all persons receiving social assistance as defined in §1 and §2 of the *Asylum Seeker Benefit Act* (AsylbLG) [40]. These are foremost persons who have a temporary residence permit as part of their request for asylum but have not yet received a decision on their refugee status (§1 par 1 Nr. 1 AsylbLG, 77% of our final sample) or whose asylum request was rejected in first instance but who received a suspension of removal (“*Duldung*”, §1 par 1 Nr. 4 AsylbLG, 14% of our final sample). Other groups are individuals with a temporary residence permit due to humanitarian reasons (§1 Abs. 1 Nr. 3 AsylbLG, 2% of our sample) and foreign nationals who are under the obligation to leave the country but have not yet done so (§1 Abs. 1 Nr. 5 AsylbLG, 3% of sample), as well as relatives of the aforementioned groups (<1%). As such, we treat the source as an almost complete census of asylum seekers (mostly at the pre-stage of becoming refugees) in Germany.

Limiting the sample to male, working-age (18 to 65) asylum seekers who reside outside of first reception centres and originate from forty-three countries of origin with the largest total numbers of asylum seekers in Germany yields a sample of approximately one million observations (see Table A1 in the S1 Appendix for a full list of countries). The focus on these forty-three countries of origin keeps 95% of asylum seekers in the sample but allows us to circumvent a potential threat to our identification strategy: asylum applicants from countries with small refugee populations tend to be assigned to areas where offices of the Federal Office for Migration and Refugees (BAMF) have regional experts [12]. We limit our analysis to male asylum seekers only as the labour market participation of female asylum seekers is influenced more heavily by unobservables [41]. Focusing on male asylum seekers does not seem to result in a stark loss of generalizability as only about a third of persons receiving asylum welfare benefits are female and they originate to a large extent from countries with low female labour market participation rates.

**Fig 3:**
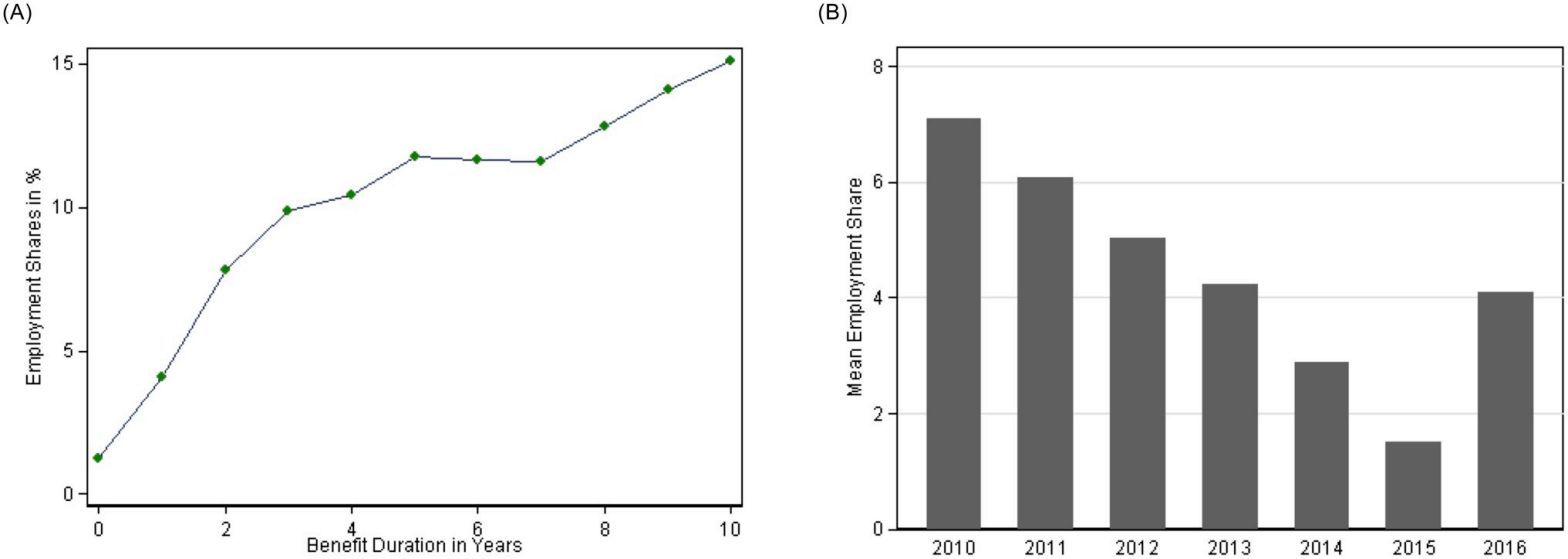
Employment shares by year and benefit duration. Panel A: Employment share by benefit duration. Panel B: Employment share by year. Source: [13], own calculations. Notes: Employment refers to any formal employment during the given calendar year. Mean is based on the sample size for that respective year.

Our main outcome of interest is employment, which takes the value of one if the individual has reported any formal employment during the calendar year and zero otherwise. Our employment definition includes self- and part-time employment, but excludes unreported informal employment arrangements by default, by that potentially under-estimating the true involvement of asylum seekers in local economic activities. Overall, on average about 3% of the asylum seekers in our sample report employment, which is extremely low (see Table A2 in the S2 Appendix for a full list of summary statistics). A strong predictor of individual employment is the time since arrival, which is reflected in Panel A of Fig 3 that plots average employment shares by benefit duration. As expected, the share of employed asylum seekers increases steadily with increasing time spent in Germany, but still stays relatively low even after a longer time horizon (whereby benefit duration is censored at 10 years). Although a large share of asylum processes gets decided within the first couple of years, the group of asylum seekers also includes individuals who receive a suspension of removal (*“Duldung”*) and stay in the country for a substantially longer time. On average, benefit duration in our sample amounts to 14 months only (see Table A2), which reflects both the fact that many asylum seekers become recognized refugees but also a composition effect as the largest number of benefit claimants entered our sample in the last two years (2015 and 2016).

It is worth noting that asylum seekers who were granted refugee status (but also those who have been most successful in the labour market) are more likely to exit the asylum seekers’ welfare benefit system over time and hence disappear from our administrative dataset. To account for this dynamic, our empirical results always control for the length of individual welfare benefit duration. Nonetheless, we can only identify current benefit claimants by the end of any given year. As can be seen from Panel B of Fig 3, employment shares varied considerably over time, first declining steadily in the period between 2010 to 2015 from initially around 7% to the record low of under 2% in 2015, while then rising again to 4% in 2016. This reflects strong composition effects, driven by the higher share of newly arrived asylum seekers in 2015, but potentially also a general overburdening of the system during this year.

We further measure job quality using information about part- and full-time employment (in the form of separate dichotomous variables). We assume full-time employment to be preferable to part-time engagements, as part-time employment often encompasses a wage penalty [42], and we expect substitution effects of wage increases to dominate potential income effects in our sample of predominantly young men with relatively low disposable income. Full-time work is recorded if the working hours in formal employment are at least equal to the regular working hours defined by the national legislation or a respective collective bargaining agreement. Although the thresholds vary by sector and occupation, usually full-time employment will involve a 35 to 40 hour working week, while part-time working hours will be significantly lower. The majority of employed asylum seekers works in a part-time contract (compare Table A2). Among those employed, about 29,800 observations also report nonzero average monthly wages, and roughly 5% of these report wages of 1500 Euros and above. This descriptive result suggests that while the majority of employed asylum seekers in the sample are in part-time and low-wage employment, a non-negligible part earns close to or above minimum wage.

**Fig 4:**
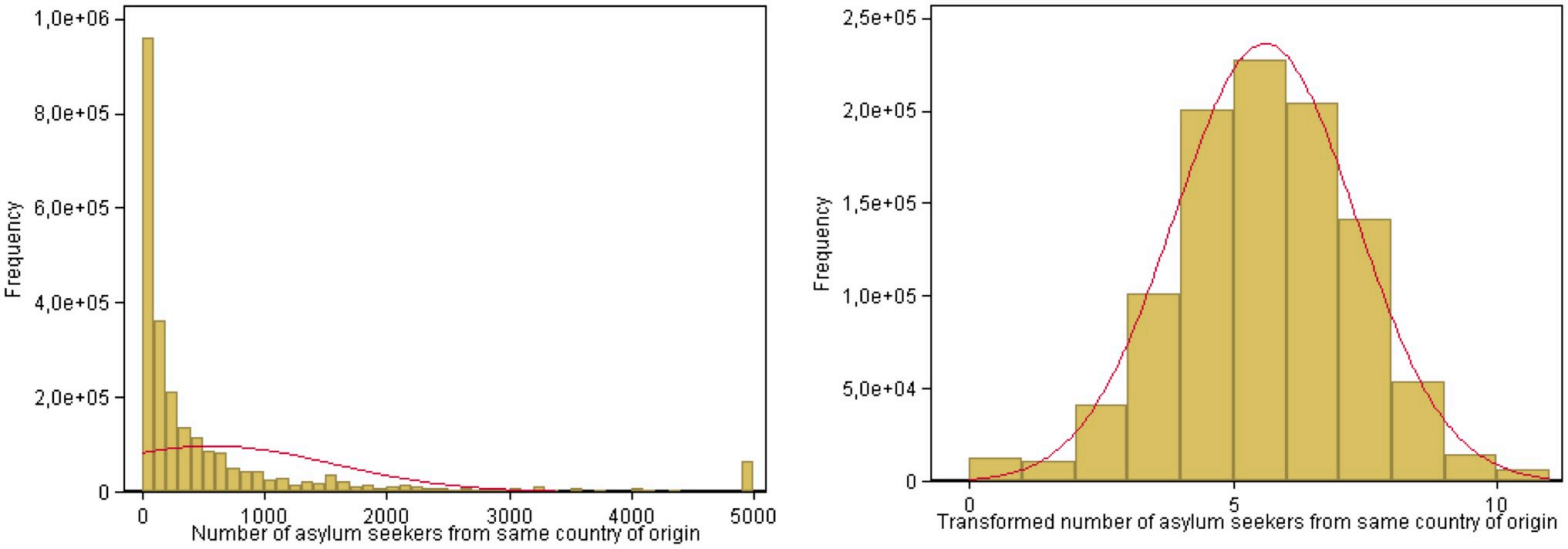
Transformed and untransformed asylum network histogram. Panel A: Untransformed network measure. Panel B: Transformed network measure. Source: [13], own calculations. Notes: In Panel A, the x-axis measures the numbers of co-national asylum seekers from the same county of origin residing in the same county, while in Panel B it refers to the inverse hyperbolic sine of the same measure. The bandwidths are set to 100 in Panel A and 1 in Panel B. In both figures, the y-axis indicates the respective frequencies, and the red line represents a normal distribution with the same mean and standard deviation.

Our main explanatory variable of interest is the size of the local co-national network of asylum seekers, based on the number of co-national asylum seekers residing in the same county within the same year. We decompose this network measure further by measuring the size of separate networks divided by employment status, cohort, and age group of the co-national asylum seekers. Cohorts are calculated using completed years of welfare benefit duration as a proxy for time since arrival. To avoid the reflection problem [43] we include separate variables for each decomposition (rather than shares) and exclude the individual asylum seeker for the calculation of his/her network measure (generating further variation within the network) [44]. We normalize our network measures taking the inverse hyperbolic sine transformation, coefficients of which can be interpreted similarly to a log function. Fig 4 plots the distributions of the transformed and untransformed asylum network variable and shows that while the untransformed measure is highly skewed to the left, the transformed measure exhibits an almost perfect log-normal distribution. While ordinary least squares estimation does not impose distributional restrictions on the independent variables, the transformed network size measure is better suited to capture decreasing marginal returns from network size to the likelihood of finding employment.

We further used data from the most recent Census to compute the number of persons with a migratory background from the same country of origin who resided in the county of the asylum seekers in 2011 [45]. This definition includes foreigners residing in Germany, German citizens who have moved to Germany after 1955, and children of at least one parent who has moved to Germany after 1955. While this variable represents a comprehensive measure of the size of the local co-national network at the beginning of our time period, it is affected by endogenous sorting of migrants to locations. Hence, we use it only in comparison to our nationality-county pair fixed effects that control for the same variation more flexibly.

### Empirical model

We investigate the impact of co-national asylum seekers’ proximity on individual employment by estimating a three-way fixed effects model using pooled individual data:

$$Y_{ijkt}=\alpha_{0}+\gamma N_{jkt}+X_{ijkt}\beta +\theta_{jt}+\vartheta_{kt}+\tau_{jk}+\epsilon_{ijkt}$$ (1)

where *Y*_*ijkt*_ is the employment probability of asylum seeker *i* of nationality *j*, residing in county *k* in year *t*. Our main variable of interest is given by *N*_*jkt*_ that measures the inverse hyperbolic sine (denoted by *asinh*) of the network size given by the number of co-national asylum seekers residing within the same county (also sub-divided by employment status, arrival cohort and age of network members). The vector of individual controls *X*_*ijkt*_ includes a set of individual and household characteristics. These include indicators for age in five-year intervals, household size, residential status, welfare benefit duration in months as a proxy for time spent in Germany, the size of non-labor income, and a set of dichotomous variables indicating whether the person is a household head, has children, resides in private accommodation, and whether their case is administered by a non-local carrier. This latter means that social registry administration lies in the responsibility of an agency at higher administrative level than the municipality, thus proxying for administrative differences between districts.

Crucially for our approach, we also control for multiple non-nested sources of variation by an extensive set of fixed effects that capture several different sources of heterogeneity in employment status and help to identify time- and location-specific variation across asylum seekers of various nationalities living within the same location, alleviating potential concerns related to a not entirely random sorting of asylum seekers to municipalities. We use in total 301 nationality-year fixed effects, *θ*_*jt*_, to factor out common Germany-wide employment fluctuations among asylum seekers coming from each of the forty-three origin countries. For instance, they factor out the on average lower probability of Syrian refugees to enter employment in Germany at the height of their influx in 2015 and 2016, as well as the effects of policy changes in 2015 that affected asylum seekers from ‘safe origin’ countries, irrespective of their place of living. A set of county-year fixed effects, *ϑ*_*kt*_, controls for idiosyncratic variation in local labour market conditions in each of the 417 counties as well as the yearly numbers of total asylum seekers assigned to a locality (factoring out a total of 2845 county-year effects). They capture all location-specific shocks over time that affect the employment probability (both from the side of labour demand and labour supply) of all asylum seekers within a given county irrespectively of their nationality. By that, they also control for remaining common selection processes that would violate a quasi-experimental allocation, like local economic factors that attract all asylum seekers irrespective of their nationality. Finally, the time invariant nationality-county pair fixed effects *τ*_*jk*_ capture nationality-specific local labour market characteristics in each county, reflecting for instance the historical presence of co-national networks. They factor out time-invariant differences across 10597 nationality-county pairs. For instance, in a county with a relatively larger Afghan diaspora, asylum seekers of Afghan origin may receive more assistance to enter the labour market than those from other nationalities in any given year. Our preferred specifications partial out this average difference through the nationality-county pair fixed effects and consider only idiosyncratic variation in Afghan asylum seekers’ employment within a county over time.

The treatment effect, *γ*, is thus given by comparing the difference in employment probabilities linked to co-national asylum seeker network size between each nationality within a county and the same year, to the difference between the effects of the same group in another county. We estimate Eq (1) by a linear probability model with three-way fixed effects [46] and cluster all standard errors at the nationality-county pair level. We refrain from using a nonlinear (probit or logit) specification despite modelling small employment probabilities as we see substantial value in implementing non-nested fixed effects for identification purposes and nonlinear models have their own weaknesses in the case of rare events, in particular the incidental parameter problem [47].

### Issues of identification

The main identification strategy of this study is based on the German dispersal policy of asylum seekers during the observation period between 2010 and 2016. We use the variation in the national composition of asylum seekers in German counties to compare the labour market outcomes of those living in areas with a higher number of co-nationals to those living in areas with a lower number of co-nationals. Consistent estimation requires the model residual to be uncorrelated with the measure of migrant networks. Our first identifying assumption is that the yearly variation in local network size is independent of unobserved characteristics of the asylum seekers. Two main institutional features are required for this assumption to hold. Firstly, the allocation decision itself must not be based on unobservable characteristics of asylum seekers. Secondly, ex-post sorting must be negligible, specifically in terms of independence from relevant unobserved characteristics. In the following, we argue that both assumptions are likely to be met to a large extent. As a second step of our identification strategy, we employ the three-way fixed effects presented above. These are needed to account for changing employment dynamics over time but can also help to alleviate concerns of endogenous sorting in case our first identifying assumption was violated.

For the purposes of identification, the allocation policy of asylum seekers within Germany provides a relatively credible (although certainly not perfect) policy experiment. As we focus on asylum seekers who are still restricted by the German asylum policies, under the assumption of quasi-random dispersal, we can directly estimate the effects of the location of co-national asylum seekers (conditional on time effects). Firstly, the centralized administration through the EASY system, which decouples the point of entry into Germany from the final place of residence, eliminates spatial dependencies between the point of entry and residence. Secondly, the administrative separation between the asylum procedure and the allocation procedure ensures that the authorities have minimal information about the asylum seekers’ background, thereby ruling out selection on unobservable characteristics. In fact, the allocation decision almost completely neglects the individual wishes of the applicant. Importantly, employment prospects and cultural proximity are not considered as substantial reasons by the authorities [19]. Lastly, the restrictions to movement as part of the domicile obligation reduce the prevalence of ex-post sorting to a minimum. In summary, we argue that both the nearly random allocation as well as the no ex-post sorting assumption can be expected to hold to a large degree because i) the asylum and the allocation procedures are administratively separated, ii) the allocation neglects asylum seekers’ preferences, and iii) the domicile and residence obligation limit sorting according to unobservable characteristics to a minimum.

To further ensure the credibility of our identification strategy, we follow the previous literature and measure the size of the co-national network at a relatively larger administrative unit. As suggested by [34] and [48], the location decision of migrants is influenced more by the number of co-nationals within different parts of a city, but less so by their presence in a larger region. In our case, we decide to measure the size of the co-national asylum seeker network at the county level, instead of the municipality level. Assuming that this measure is still a valid proxy of co-national ties, this approach ensures identification while allowing for ex-post sorting within counties [49]. In view of previous results, we believe that measuring the network at the county level offers a valid approach. For example, [50] investigate the effects of the network with regard to proximity and find significant positive network effects for up to one hour travel time. According to the results from the last census in 2011, the median size of the 431 German counties was around 800 km^2^, which should be well traversed within an hour. This means that we expect network effects to be found at the county level and not only at the municipality level.

Beyond the main specification that focuses on the local size of the co-national asylum seeker network, we further decompose the network size variable according to characteristics of the network members, relying on the time since arrival, employment status and age of the co-national asylum seekers. The strength of identification varies across these decompositions. Whereas due to the placement policies, we consider the number of co-national network members as exogenously given in any location, this is not necessarily true to the same extent for all decomposed measures. A decomposition of network size by cohort of arrival or age requires that the dispersal policy does not condition the placement of asylum seekers on their own characteristics (age) or on placement history (arrival cohort size). To the best of our knowledge, this has not been the case in Germany and hence the results on network size by age or arrival cohort may still offer some credibility. However, our decomposition of network size by employment status is conditioning on a variable that is in itself endogenous as it responds to local economic conditions. Thus, results on the network size by employment status should be interpreted with more caution and only conditionally on the county-year fixed effects that control for county-level labor market dynamics.

One potential drawback of the registry data is that it contains only limited individual information. If some refugees manage to circumvent spatial assignment, the employment regressions may suffer from omitted variable bias by not controlling for relevant determinants of individual labour market success (like education) if these are correlated with the local network size. For instance, if less educated asylum seekers value the presence of co-nationals more strongly, they will try to self-select into places with larger co-national asylum seeker networks. Random assignment of asylum seekers across German counties would fully deal with these concerns. If it is violated, using nationality-year fixed effects allows us to control at least for time-varying differences in educational attainment between asylum seekers of different nationalities and especially composition effects arising from general nationality-specific shifts of refugee flows over time. Although there is no evidence for substantial self-selection of asylum seekers into locations in Germany, we cannot exclude this concern entirely.

A further limitation of this study lies in the cross-sectional nature of the data sources, which means that we are not able to follow the same asylum seekers over time. Issues of sample selection can potentially affect our results as asylum seekers graduate out of the sample after successful asylum applications, while asylum seekers whose asylum claims are rejected in first instance but their removal is suspended (‘*Duldung’*) may continue receiving asylum welfare benefits. To account for this dynamic, our empirical results always control for the length of individual welfare benefit duration in months. Nonetheless, we can only identify current benefit claimants in any given year and do not consider recognized refugees.

As a result, our network measure only captures yearly variation in the number of locally resident asylum seekers but not in the number of recognized refugees or other co-national migrants. This is due to limitations on the administrative data as there is no publicly available county-level information on the yearly number of refugees by origin nationality in Germany. The use of asylum seeker benefit registry data provides us with a unique (and hitherto unused) opportunity to generate location-specific time-variant measures of network size. Moreover, when focusing on asylum seekers we can avoid issues of a selection into locations to a large extent (due to the dispersal policies in place) whereas the same cannot be claimed for refugees before 2016, nor for migrants. However, focusing on co-national asylum seekers comes at the cost that we substantially underestimate the total number of co-nationals living in the same county. Hence, our network measure is not able to capture the full importance of already better established migrant networks, and focuses on the short-run importance of early networks among the co-national asylum seekers.

Our use of fixed effects can partially help to relieve some of the concerns arising from this limitation. We expect that co-nationals who have been already better established within the locality may help individual employment by providing information or direct assistance to asylum seekers to access the labour market. Thus, asylum seekers of different nationalities may face on average different labour market conditions within the same local labour market. Including nationality-county pair fixed effects will control for within-county variation in the historical size of the co-national migrant (and former refugee) network. Conditional on local labour market shocks (which are fully captured by county-year fixed effects), this will factor out time variant differences due to the historical network size. Comparing coefficients from estimations with and without nationality-county pair fixed effects will also provide suggestive evidence on the potential importance of historical migrant networks for explaining fluctuations in individual employment. However, this still cannot control for the most recent variation in the size of the co-national local refugee network and thus our setting is less suited to investigate full network effects by arrival cohort.

## Results

Table 1 reports the network coefficients from regressions on the probability of employment. In both panels, control variables are added consecutively, first including individual-level controls (column (2)) and extending the specifications by further non-nested fixed effects. Column (5) reports our preferred full specification as described in Eq (1).

**Table 1:** Employment effects of co-national networks of asylum seekers.

Dependent Variable	Employment					
	(1)	(2)	(3)	(4)	(5)	(6)
*Panel A*						
*asinh* Network size	-0.005***	-0.002**	0.000	0.001	0.000	0.000
	(0.001)	(0.001)	(0.001)	(0.001)	(0.001)	(0.001)
*asinh* Initial migrant network size						0.004***
						(0.001)
*Panel B*						
*asinh* Employed network size	0.026***	0.022***	0.023***	0.021***	0.013***	
	(0.001)	(0.001)	(0.001)	(0.001)	(0.001)	
*asinh* Unemployed network size	-0.015***	-0.011***	-0.013***	-0.012***	-0.009***	
	(0.000)	(0.000)	(0.001)	(0.001)	(0.001)	
Controls	No	Yes	Yes	Yes	Yes	Yes
Nationality-Year FEs			Yes	Yes	Yes	Yes
County-Year FEs				Yes	Yes	Yes
Nationality-County Pair FEs					Yes	
No. observations	1014790	985868	985868	985850	984801	934831

Source: [13], own calculations. Note: The dependent variable measures the individual probability of reporting employment. Network size is measured by the inverse hyperbolic sine of the number of co-national asylum seekers residing within the same county in the same year and is further split by the co-nationals’ employment status in panel B. The initial migrant network size in each county is derived from Census 2011 and refers to the total number of co-nationals living within the county in 2011. Controls include indicators for age in five-year intervals, household size, residential status, welfare benefit duration, the size of non-labor income, non-local support facility, and indicators for parents and household heads. Standard errors in parentheses are clustered at the nationality-county level. Significance levels * p < 0.1, ** p < 0.05, *** p < 0.01.

In column (1) of Panel A, we see a negative correlation between local network size and the likelihood of employment. With a 100 asinh (or log) points increase in local network size (which would more than double the network size), the individual probability of employment declines by 0.5 percentage points, or by about 17% of the average employment in the sample (which is 0.03). This negative correlation is more than halved once we control for observed individual characteristics of asylum seekers in column (2) but it still stays significantly negative. However, as soon as we control for common country-wide employment dynamics of asylum seekers of different nationalities by including a full set of country-of-origin nationality by year fixed effects in column 3, the negative relationship completely vanishes. This indicates that the negative correlation between local network size and employment is purely driven by time dynamics in new arrivals of asylum seekers to Germany from different locations. Adding further controls for county-specific variation in labor market conditions over time (in column (4)) as well as nationality-county-specific average differences in column (5) does not change this result. In places where and in times when the number of local co-national asylum seekers is high, fewer of them are able to enter the German labor market but this can be fully explained by general national trends and does not depend on the local size of the co-national network itself. As a further check, column (6) replaces the nationality-county fixed effects and controls directly for the number of co-national migrants residing within the same county in 2011 instead. This information is derived from the German Census 2011 and it shows a positive correlation between the number of co-nationals in a county and early labor market entry of asylum seekers just as predicted by the information channel. When increasing the size of the initial migrant network by 100 asinh-points, the individual likelihood of employment increases by 0.4 percentage points. Nonetheless, as we want to focus on the short-run effects of the asylum seeker network which we expect to be subject to more idiosyncratic variation, for the further results we will retain column (5) as our favorite specification and will factor out the potential importance of the more established migrant networks.

More co-national asylum seekers in the same county imply a larger network with presumably positive employment effects, but also an increase in the nationality-specific labor supply, increasing competition for information within the same network. We study this relationship by splitting the composite network measure by employment status in Panel B of Table 1, and by cohort, and age group in Table 2, and find significant compositional heterogeneities.

**Table 2:** Employment effects: Heterogeneity by arrival and age cohorts.

Dependent Variable	Employment			
	(1)	(2)	(3)	(4)
*Panel A asinh* Network size by cohort of arrival:				
Same cohort	-0.009***	-0.001***	-0.001***	-0.001***
	(0.000)	(0.000)	(0.000)	(0.000)
*t-1*	-0.001**	0.000	0.000	0.000
	(0.000)	(0.000)	(0.000)	(0.000)
*t-2*	0.002***	0.001**	0.001*	-0.000
	(0.000)	(0.000)	(0.000)	(0.000)
*t-3*	0.002***	0.001*	0.000	-0.000
	(0.000)	(0.000)	(0.000)	(0.000)
*t-4*	0.002***	0.001	0.001	0.000
	(0.001)	(0.000)	(0.000)	(0.001)
*Panel B asinh* Network size by age group:				
0–18	-0.003**	0.002**	0.001*	-0.000
	(0.001)	(0.001)	(0.001)	(0.001)
18–25	-0.006***	-0.003***	-0.002***	-0.004***
	(0.001)	(0.001)	(0.001)	(0.001)
26–35	-0.007***	-0.004***	-0.003***	-0.001
	(0.001)	(0.001)	(0.001)	(0.001)
36–65	0.006***	0.004***	0.003***	0.005***
	(0.001)	(0.001)	(0.001)	(0.001)
> 65	0.007*	0.004**	0.004**	0.002*
	(0.004)	(0.001)	(0.002)	(0.001)
Controls	No	Yes	Yes	Yes
Nationality-Year FEs		Yes	Yes	Yes
County-Year FEs			Yes	Yes
Nationality-County Pair FEs				Yes
No. observations	1014790	985868	985850	984801

Source: [13], own calculations. Note: The dependent variable measures the individual probability of reporting employment. Network size is measured by the inverse hyperbolic sine of the number of co-national asylum seekers residing within the same county in the same year and is further split by the co-nationals’ cohort of arrival and age. Controls include indicators for age in five-year intervals, household size, residential status, welfare benefit duration, the size of non-labor income, non-local support facility, and indicators for parents and household heads. Standard errors in parentheses are clustered at the nationality-county level. Significance levels * p < 0.1, ** p < 0.05, *** p < 0.01.

The most important of these heterogeneities is by network quality, measured as employment within the network (Panel B of Table 2): Individual employment probability increases significantly with the number of employed co-nationals and decreases with the number of non-employed co-nationals living within the same county.

Without the inclusion of any controls (column 1), the positive correlation of individual employment probability with the number of employed asylum seekers in the county (0.026) is almost twice as large as the negative correlation with the number of non-employed co-nationals (-0.0l5). For individual employment probabilities, it seems to be more beneficial to have a proportionate increase in the number of working co-national network members within the county than it is harmful to have a similar proportionate increase in non-working co-nationals. However, these correlations do not necessarily reflect a causal relationship. Even if the assignment of asylum seekers was random, their employment is going to reflect changes in local demand conditions, shifting our focus even more strongly to our fixed effects specifications. Coefficients decrease only slightly when introducing a set of individual-level controls in column 2. Moreover, unlike in the case of the total network size in panel A, local network size by employment status stays significant throughout all fixed effects specifications. Neither controlling for nationality-specific employment dynamics in column (3), nor for county-year fixed effects in column (4) change the estimated relationship substantially. Hence, these results are not driven by common nation-wide employment trends affecting asylum seekers of different nationalities, nor by within-county changes in labor market conditions or local policies over time that would affect all asylum seekers in the same way.

Finally, column 5 includes fixed effects for each nationality-county pair. By that, it controls for time-invariant differences between the employment likelihood of asylum seekers of various nationalities in each county, reflecting especially the role of the historical migrant networks from the same country of origin, but also other idiosyncratic county-specific differences in the systemic ability of supporting asylum seekers of different nationalities. Indeed, controlling for nationality-county pair fixed effects reduces estimated coefficient sizes further. This conforms with our expectation that historical migrant networks also contribute to facilitating labor market access of newly arrived asylum seeker co-nationals. In column (5), an increase in the number of employed co-national network members by 100 asinh points (or by approximately 170%) leads to an increase in the probability of employment by 1.3 percentage points. This corresponds to an increase of about 40% relative to the sample mean employment rate. At the same time, asylum seekers’ likelihood of employment declines by about 0.9 percentage points when the number of non-employed co-national asylum seekers in the county increases by 100 asinh points.

Of course, if local labor demand fluctuations are fully nationality specific, this result could also reflect unobserved local labor demand shocks for employees from certain nationalities. However, we doubt that German county-level labor markets are segregated across each of the origin countries of asylum seekers to such an extent as to render yearly fluctuations of local demand fully nationality specific. We think that it is more likely that our measures of the number of (non-)employed network members will also reflect nationality-specific local supply conditions. This result thus shows that living in areas where more co-nationals are in employment is helping the individual labor market success, whereas being surrounded by additional non-employed network members reduces the individual likelihood of work. Both the positive and the negative effects may arise from differences in the access to and competition for information as well as the role of social norms.

Splitting the co-national network based on their time of arrival in Germany in Table 2 (using welfare benefit duration as proxy for time since arrival), we find only weak evidence for cohort-based heterogeneities among asylum seekers as documented by [10] for refugees. The unconditional model (column (1) of Panel A) displays the expected pattern to some extent: The likelihood of employment reduces with the size of the own cohort of co-national arrivals, while cohorts that have arrived two years before (but have still not received refugee status) increase the probability of employment among the more recently arrived, thus pointing towards competition dominating at the beginning, whereas positive effects appearing over time. However, the relatively small effect sizes practically vanish once we introduce a fuller set of controls in columns (2) to (4). Only the number of co-nationals arrived within the same year is still significantly negatively linked to individual employment, but the effect is negligible in size. As discussed before, these results do not mean that co-national migrant networks are not beneficial. The historical size of co-national networks is factored out by the nationality-county pair fixed effects. As we cannot observe fluctuations in the number of co-national refugees within the same county, we cannot exclude that beneficial information effects are provided by the recently established refugees instead of the asylum seekers.

Finally, we also find partial evidence for heterogeneities in the age composition of the network (Panel B of Table 2). Being surrounded by additional young (old) co-nationals inhibits (improves) employment prospects. These results are conditional on the individuals’ own age and suggest that young and old network members may play different roles in providing labor market information. Whereas the younger may act as competitors in the labor market, older network members may function more as mentors. However, in terms of effect sizes, individual employment outcomes respond much more strongly to the number of employed co-national members than to the numbers belonging to different cohorts by the time of arrival or age. Table 3 reports the network coefficients from regressions on the probability of full- and part-time employment. In each panel, the left column reports regressions using the dichotomous outcome of part-time employment, while the right panel uses the full-time employment as dichotomous outcome. All results are based on the full specification as in Eq (1), controlling for nationality-year, county-year, and nationality-county-pair fixed effects.

**Table 3:** Part- and full-time employment effects of co-national networks.

Dep. Variable: Employment							
	Part-time		Full-time			Part-time	Full-time
Panel A				Panel B			
Network Size		0.001	-0.001*	Network Size of Employed		0.011***	0.007***
		(0.001)	(0.001)			(0.001)	(0.000)
				Non-employed		-0.005***	-0.005***
						(0.000)	(0.000)
Panel C				Panel D			
Network Size by Cohorts				Network Size by Age			
	same	-0.001***	-0.001***		0–18	-0.001**	0.000
		(0.000)	(0.000)			(0.000)	(0.000)
	t-1	0.000	0.000		19–25	-0.001**	-0.001**
		(0.000)	(0.000)			(0.001)	(0.000)
	t-2	0.000	0.000		26–35	-0.001**	0.000
		(0.000)	(0.000)			(0.001)	(0.001)
	t-3	0.000*	0.000		36–65	0.001*	0.001
		(0.000)	(0.000)			(0.001)	(0.000)
	t-4	0.001***	-0.001**		> 65	0.004***	-0.001*
		(0.000)	(0.000)			(0.002)	(0.000)
Further controls		*Yes*	*Yes*			*Yes*	*Yes*

Source: [13], own calculations. Note: Definitions of the dependent variable and controls are described in the text. All regressions include a full set of controls and nationality-year, county-year and nationality-county pair fixed effects. Standard errors in parentheses are clustered at the nationality-county pair level. N = 984,801 in panel A, N = 985,850 in panel B and C, and N = 958,144 in Panel D. Significance levels * p < 0.1, ** p < 0.05, *** p < 0.01.

These results are in line with what we found when using the overall employment indicator, but add some interesting new detail. In Panel A we find a very small and marginally significant negative association between the size of the local co-national network and the likelihood of being in full-time employment, while the association between asylum seeker network size and part-time employment is insignificant.

The previously emphasized importance of network quality is confirmed in Panel B, replicating the link between having more employed co-national asylum seekers and a larger probability of employment, together with negative employment effects from having more non-employed co-national asylum seekers living nearby. Interestingly however, the effect sizes vary considerably depending on the outcome used. An increase in the number of employed co-national network members by 100 asinh points leads to an increase in the probability of part-time employment by 1.1 percentage points as compared to 0.7 percentage points for full-time employment. An intuitive explanation for this result is that even when networks are successful at increasing employment, they do so mostly in lower quality jobs. This explanation has been supported by previous studies in the literature [51, 52]. However, we would refrain from making strong causal claims in this regard. Firstly, our measure of job quality is only a rough approximation of job quality measured in terms of wages and/or job satisfaction. Secondly, due to bureaucratic constraints, it is likely considerably easier for asylum seekers to work in part-time employment. In Panel C, when splitting the network measure by cohort size, we again cannot confirm the pattern predicted by a dynamic cohort model, neither for part- nor for full-time employment. Regarding the age decomposition in Panel D, the effect sizes are considerably smaller, and again only significant for part-time employment.

## Conclusion

In this paper, we associated variations in the size of local co-national asylum seeker networks to the employment probability of asylum seekers in Germany. We argued that the natural experiment created by the German refugee dispersal policy, when accompanied by an extensive fixed effects strategy, allows us to establish an empirical link between total network size and employment, also when differentiated by pre-determined individual characteristics of network members (like age). Our identification strategy conditions all results on county-year, origin nationality-year and also nationality-county pair fixed effects, which help to come closer to identifying the effects of co-national asylum seekers on individual employment even in case of a violation of quasi-random assignment of asylum seekers across Germany. Moreover, under the assumption that yearly fluctuations in local labor demand are not fully nationality specific, we can also link the number of employed network members to own employment.

Our results are based on hitherto little used German registry data of asylum seekers, which, although highly informative, also brings some limitations for the empirical strategy. Specifically, the registry data does not allow for following individuals over time and does not include the already well-integrated accepted refugees who have become entitled to other forms of social assistance, or, decided not to claim any assistance at all. Thus, our results do not capture a full co-national network effect, but focus on the importance of co-national networks among the most recently arrived asylum seeker cohorts. As early labor market integration has often been considered as highly relevant for long-term integration of refugees, our finding of relatively limited negative effects from co-location of co-national asylum seekers could still help to inform future asylum dispersal policies.

The presented results align well with previous research and add some interesting new insights to this well-established research question. In line with previous studies from Germany [39, 53], we find an insignificant composite effect of co-national networks on employment probability and only a weak negative effect on full-time employment. Conditional on local employment prospects, asylum seekers do not seem to be quicker at finding work if surrounded by co-national asylum seekers and are substantially less likely to be employed when the number of non-employed co-national asylum seekers rises. Taken together, this may indicate that the optimistic findings from the U.S. literature on the beneficial effects of co-ethnic networks in general may not be applied directly to the context of the short-run effects of co-location of asylum seekers in Germany.

We also tested for network composition effects by splitting our network measure by employment, cohort, and age. The results underline the central importance of network quality. Having more co-national asylum seekers who are actually in formal work, helps individual employment substantially. By contrast, increasing the number of co-national asylum seekers who are out of work, hinders individual employment prospects. Although these results may also partly reflect idiosyncratic nationality-specific local labor demand shocks over time, we believe that network quality must also contribute to this differential result. Comparing this canonical explanation with the more recent model by [10], we found that the network quality transmission channel is more fitting to the German case. In our preferred specifications, the presence of more co-national asylum seekers who have lived in Germany longer did not increase employment prospects in our sample significantly. However, we could not exclude that asylum seekers’ employment prospects may have benefited from the presence of earlier refugee cohorts and documented a positive correlation between asylum seekers’ employment and the initial network size of co-national migrants residing within the same county. Experience measured in terms of the number of older co-national asylum seekers showed significant positive impacts, albeit less strongly so than the number of employed asylum seekers. We also provided indicative evidence that migrant networks have stronger effects on the likelihood of finding part-time employment, as compared to full-time employment.

## Supporting information

S1 Appendix (DOCX)

S2 Appendix (DOCX)
