# Supplementary material for: The impact of co-national networks on asylum seekers’ employment: Quasi-experimental evidence from Germany
Source: PLoS One. 2020 Aug 4;15(8):e0236996. doi: 10.1371/journal.pone.0236996 (PMC7402491; doi:10.1371/journal.pone.0236996)
Supplement: S1 Appendix — (DOCX) [file pone.0236996.s001.docx]

**Table A1: Sample Countries of Origin**

| Country of origin | | Number of Observations | Share in % | Country of origin | | Number of Observations | Share in % |
| --- | --- | --- | --- | --- | --- | --- | --- |
| 1. | Syria | 206000 | 20.30 | 23. | Georgia | 9894 | 0.98 |
| 2. | Afghanistan | 130369 | 12.85 | 24. | Armenia | 9784 | 0.96 |
| 3. | Iraq | 90525 | 8.92 | 25. | Bangladesh | 9658 | 0.95 |
| 4. | Pakistan | 66209 | 6.52 | 26. | Bosnia & Herzegovina | 9645 | 0.95 |
| 5. | Iran | 45833 | 4.52 | 27. | Ghana | 8954 | 0.88 |
| 6. | Eritrea | 39033 | 3.85 | 28. | Ethiopia | 8807 | 0.87 |
| 7. | Serbia | 32387 | 3.19 | 29. | Egypt | 7956 | 0.78 |
| 8. | Somalia | 28157 | 2.77 | 30. | Senegal | 6170 | 0.61 |
| 9. | Albania | 27570 | 2.72 | 31. | Ukraine | 5823 | 0.57 |
| 10. | Kosovo | 25100 | 2.47 | 32. | Tunisia | 5790 | 0.57 |
| 11. | India | 25052 | 2.47 | 33. | Mali | 5090 | 0.50 |
| 12. | Nigeria | 23287 | 2.29 | 34. | Sri Lanka | 4259 | 0.42 |
| 13. | Russia | 23000 | 2.27 | 35. | Côte d`Ivoire | 4170 | 0.41 |
| 14. | Turkey | 21543 | 2.12 | 36. | Montenegro | 3869 | 0.38 |
| 15. | Lebanon | 20077 | 1.98 | 37. | Libya | 3732 | 0.37 |
| 16. | Algeria | 19093 | 1.88 | 38. | Sierra Leone | 3482 | 0.34 |
| 17. | Macedonia | 16502 | 1.63 | 39. | Benin | 3161 | 0.31 |
| 18. | Gambia | 15709 | 1.55 | 40. | Togo | 1883 | 0.19 |
| 19. | Morocco | 12179 | 1.20 | 41. | Congo, DRC. | 1417 | 0.14 |
| 20. | Azerbaijan | 10807 | 1.06 | 42. | Kenia | 1356 | 0.13 |
| 21. | Guinea | 10332 | 1.02 | 43. | Angola | 897 | 0.09 |
| 22. | Sudan Republic | 10202 | 1.01 |  |  |  |  |

Source: Source: [13], own calculations. Notes: The numbers refer to the sample of male asylum seekers, aged 18 – 65, from the chosen countries of origin. Share in % refers to the total sample size given these restrictions.
