# Supplementary material for: The impact of co-national networks on asylum seekers’ employment: Quasi-experimental evidence from Germany
Source: PLoS One. 2020 Aug 4;15(8):e0236996. doi: 10.1371/journal.pone.0236996 (PMC7402491; doi:10.1371/journal.pone.0236996)
Supplement: S2 Appendix — (DOCX) [file pone.0236996.s002.docx]

**Table A2: Summary Statistics**

| Variable Name | | Mean | Standard Deviation | Number of Observations |
| --- | --- | --- | --- | --- |
| Employment | | 0.03 | 0.13 | 1014790 |
| Full-time employment | | 0.01 | 0.11 | 1014790 |
| Part-time employment | | 0.02 | 0.13 | 1014790 |
| Age | | 30.02 | 9.80 | 1014790 |
| Benefit duration in months | | 14.62 | 24.29 | 1014790 |
| Non-labour income in € | | 7.71 | 93.12 | 1014790 |
| HH size | | 1.71 | 1.46 | 1014790 |
| HH head | | 0.89 | 0.32 | 1014790 |
| Parent | | 0.18 | 0.38 | 1014790 |
| Private accomodation | | 0.46 | 0.50 | 1014790 |
| Non-local support facility | | 0.15 | 0.36 | 1014790 |
| Residential status | |  |  |  |
|  | Residency authorization | 0.02 | 0.13 | 1014790 |
|  | Temporary residence permit | 0.77 | 0.42 | 1014790 |
|  | Return obligation | 0.03 | 0.18 | 1014790 |
|  | Family member | 0.00 | 0.06 | 1014790 |
|  | Tolerated | 0.14 | 0.35 | 1014790 |
|  | Entry via airport | 0.00 | 0.05 | 1014790 |
|  | Subsequent asylum application | 0.01 | 0.09 | 1014790 |
| No. asylum seekers | | 4360.80 | 8340.19 | 1014790 |
| No. co-national asylum seekers | | 526.03 | 1452.46 | 1014790 |
| No. employed co-national asylum seekers | | 7.50 | 30.84 | 1014790 |
| No. not-employed co-national asylum seekers | | 518.53 | 1446.50 | 1014790 |
| No. co-national asylum seekers age below 18 | | 158.26 | 449.91 | 1014790 |
| No. co-national asylum seekers age 18 -25 | | 150.53 | 420.78 | 1014790 |
| No. co-national asylum seekers age 26 -35 | | 123.75 | 342.28 | 1014790 |
| No. co-national asylum seekers age 36 -65 | | 89.01 | 244.47 | 1014790 |
| No. co-national asylum seekers age over 65 | | 4.48 | 18.37 | 1014790 |
| Cohort size co-national asylum seekers | | 360.79 | 1212.14 | 1014790 |
| t-1 Cohort size co-national asylum seekers | | 50.09 | 209.97 | 1000680 |
| t-2 Cohort size co-national asylum seekers | | 10.26 | 36.33 | 996988 |
| t-3 Cohort size co-national asylum seekers | | 4.32 | 19.03 | 992520 |
| t-4 Cohort size co-national asylum seekers | | 2.36 | 12.88 | 987075 |

Source: [13], own calculations. Notes: Non-labour income is set to zero if no other income sources were reported. Numbers of asylum seekers refer to the number in each county at each year. Cohort lags are calculated based on welfare benefit duration in years up to a maximum of ten years. For those who by construction cannot have a lagged cohort (e.g. with ten years welfare benefit duration) the measure is set to missing.
